# Three-dimensional magnetic resonance imaging of fetal head molding and brain shape changes during the second stage of labor

**DOI:** 10.1371/journal.pone.0215721

**Published:** 2019-05-15

**Authors:** Olivier Ami, Jean Christophe Maran, Petra Gabor, Eric B. Whitacre, Dominique Musset, Claude Dubray, Gérard Mage, Louis Boyer

**Affiliations:** 1 Ramsay Generale de Sante, La Muette Clinic, Paris, France; 2 Image Guided Therapies, Pascal Institute, UCA CNRS SIGMA, University of Clermont Auvergne, Clermont-Ferrand, France; 3 Department of Obstetrics and Gynecology, Hospital Center of Argenteuil, Argenteuil, Île-de-France, France; 4 Breast Center of Southern Arizona, Tucson, AZ, United States of America; 5 Paris-Sud University Faculty of Medicine, Le Kremlin-Bicetre, Île-de-France, France; 6 Center for Clinical Research, University of Auvergne, Clermont-Ferrand, France; 7 Department of Obstetrics and Gynecology, University of Auvergne, Clermont-Ferrand, France; 8 Department of Radiology, CHU Clermont-Ferrand Gabriel Montpied, Clermont Ferrand, France; 9 GIE IMIDF, Clinique de l'Essonne, Evry, France; Brigham and Women's Faulkner Hospital, UNITED STATES

## Abstract

To demonstrate and describe fetal head molding and brain shape changes during delivery, we used three-dimensional (3D) magnetic resonance imaging (MRI) and 3D finite element mesh reconstructions to compare the fetal head between prelabor and the second stage of labor. A total of 27 pregnant women were examined with 3D MRI sequences before going into labor using a 1 Tesla open field MRI. Seven of these patients subsequently had another set of 3D MRI sequences during the second stage of labor. Volumes of 2D images were transformed into finite element 3D reconstructions. Polygonal meshes for each part of the fetal body were used to study fetal head molding and brain shape changes. Varying degrees of fetal head molding were present in the infants of all seven patients studied during the second phase of labor compared with the images acquired before birth. The cranial deformation, however, was no longer observed after birth in five out of the seven newborns, whose post-natal cranial parameters were identical to those measured before delivery. The changing shape of the fetal brain following the molding process and constraints on the brain tissue were observed in all the fetuses. Of the three fetuses presenting the greatest molding of the skull bones and brain shape deformation, two were delivered by cesarean-section (one after a forceps failure and one for engagement default), while the fetus presenting with the greatest skull molding and brain shape deformation was born physiologically. This study demonstrates the value of 3D MRI study with 3D finite element mesh reconstruction during the second stage of labor to reveal how the fetal brain is impacted by the molding of the cranial bones. Fetal head molding was systematically observed when the fetal head was engaged between the superior pelvic strait and the middle brim.

## Introduction

Humans have a pelvis that is less conducive to easy delivery of the large fetal head than other mammals. Factors related to a safe vaginal delivery include an adequately sized and shaped maternal bony pelvis, soft tissue shaping of the birth canal during delivery, sufficient uterine contractions, and a fetal head of the proper size and ability to mold. Labor dystocia most often results from a combination of these fetal and maternal factors. Even when vaginal delivery is possible, the birth process can be traumatic to the infant, with asymptomatic brain hemorrhages[1] and retinal hemorrhages[2] occurring in up to 43% of vaginally delivered neonates [3].

A minor degree of pelvic dystocia, asynclitism and molding of the fetal head can precipitate a difficult vaginal delivery [4]. A prediction before onset of spontaneous or induced labor of the final route of delivery–vaginal or cesarean (C)-section–is desired by mother and physician.[5]. Unfortunately, most pelvic dystocia cases are not diagnosed until labor is well advanced. Little is known about the possible causes or mechanisms of labor dystocia and brain and retinal hemorrhage. Predictive models, such as the three-dimensional (3D) birth simulation software Predibirth (Olivier Ami, France), require study of the process of birth with 3D imaging [6].

With this in mind, we performed sequential Magnetic Resonance Imaging (MRI) imaging and 3D reconstruction of the fetal skull and brain in a series of normal pregnancies with planned vaginal deliveries before the onset of labor and then during the second stage of labor. The study’s aim was to describe fetal head molding during the birthing process.

## Materials and methods

Seven pregnant women were examined with 3D MRI before going into labor and during the second stage of labor [6]. This prospective biomedical observational study (IMAGINAITRE) was approved by the French ethical Institutional Review Board "Ile de France II" (ID-RCB 2012-A01469-34) and by the French National Agency for Drug and Medical Product Safety (*Agence Nationale de Sécurité du Médicament et des Produits de Santé*) and promoted by the University of Clermont-Ferrand Medical Center. All the women agreed to participate and signed an informed consent.

### Clinical protocol

Women recruited for the study were pregnant, aged 21–39 years, had an effective epidural during labor, were primipara, secundipara or tertipara, and had no known factors that could impair vaginal delivery. Exclusion criteria included not strictly cephalic presentation at the time of delivery, scarred uterus, more than three prior pregnancies, maternal or fetal medical conditions known to require or requiring urgent care, abnormal fetal heart rate requiring treatment within half an hour, contraindications to MRI, a minor or incompetent adult patient, no prior obstetrical care during pregnancy, or a Magnin score less than 22 cm.

Pre-labor MRI was performed between 36 and 39 weeks of gestation. Imaging during the second stage of labor was performed no more than 10 minutes before the onset of expulsive efforts. Entry into the second phase of labor was marked by complete cervical dilation accompanied by descent of the fetal head, with engagement between the superior pelvic strait and the middle of the pelvic brim verified by clinical examination. The patients were imaged in the dorsal decubitus position for both studies and were at rest during the MRI scans, just before pushing. The attending obstetrician examined the newborn cranial anatomy for the presence of molding.

Special considerations were made for the safety of mother and child as previously described[6]. Briefly, patients were cared for during the study by dedicated teams consisting of an obstetrician, radiologist, midwife, and pediatrician. Patient transportation time from the MRI suite to the delivery room in the same building, bed to bed, was less than 3 minutes. All patients received the same attention, monitoring and standard of safety as patients undergoing routine delivery.

### Patient population

The physical characteristics of these seven women and their birthing processes have been described [6]. The women were 23–34 years of age, beyond 39 weeks gestation, and had a Magnin score of 25.1 to 27.5. The fetuses descended a range of 23.1 to 53.8 mm and had an angle of progression ranging from 17. 6° to 48. 5°, according to Dietz et al.[7]. Membranes were ruptured in 6 of the 7 women.

Newborn weight ranged from 3095 to 4525 g. One patient had a 1-minute Apgar score of 4 and all the other 1-minute and all 10-minute Apgar scores were 10. Five children were born by vaginal delivery without instrumentation and two by emergency C-section (one of these after forceps failure).

### Imaging protocol

The same protocol was used for the tests, both before and during the second stage of labor, with the same sequences, the same coils, and the same conditions of maternal position.

The sequences used were fast and contiguous, lasting approximately 30 seconds each, without any contrast injection. The duration of data acquisition never exceeded 12 minutes.

The protocol included a localization sequence, acquisition of contiguous frontal, sagittal and axial T1-weighted, 3D gradient echo, as well as a T2-weighted acquisition of fetal brain volume and a dynamic acquisition for one minute sagittally centered on the birth canal.

- 3D T1 sequence for bone imaging (e-THRIVE, Philips) single shot sequence without fat saturation (FAT-SAT); echo time (TE), 1.62 ms; repetition time (TR), 3.2 ms; matrix, 448x448 pixels; angle, 10; turbo field echo (TFE) factor, 82; number of signal averages (NSA), 1; 160 overlapping sections; acquisition voxel size, 2.2 mm; reconstructed voxel size, 1 mm.
- T2 breath-hold sequence for the fetal brain (Balanced Fast Field Echo, Philips) spin echo sequence without FAT-SAT; TE, 120 ms; TR, 831 ms; Matrix, 432x432 pixels; angle, 10; TFE factor, 82; NSA, 1; 100 overlapping sections; Acquisition voxel size, 0.91 mm; Reconstructed voxel size, 1 mm.
- T1 dynamic sagittal sequence (Dynamic Balanced Fast Field Echo, Philips) single shot sequence without FAT-SAT; TE, 1.65 ms; TR, 3.29 ms; Matrix, 448x448 pixels; angle, 45; TFE factor 82; NSA, 1; 100 overlapping sections; acquisition voxel size, 1.17 mm; reconstructed voxel size, 1 mm.

The imaging was performed in an open 1-T Philips Panorama MRI, located in the same building as the maternity ward.

### Image analysis

The image analyses were performed by two radiologists experienced in the field of perinatal MRI. Inter-observer agreement was obtained after a discussion of the findings.

Images and 3D reconstructions were reset to fixed points of the maternal pelvis (acetabulum, pubis, iliac crests, the promontory of the sacrum, coccyx) in order to facilitate comparison.

**Fig 1:**
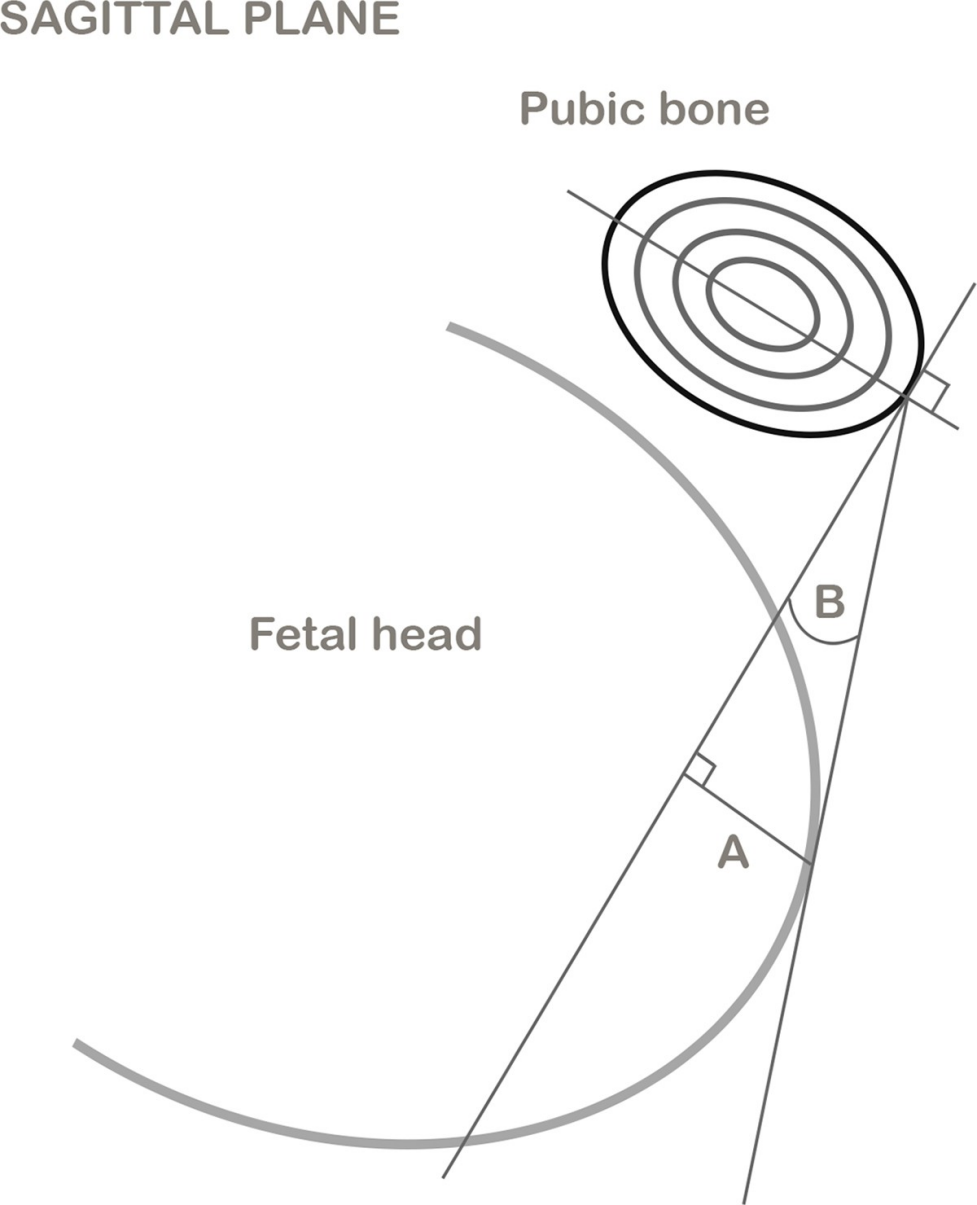
Measure of the height reached by the fetal presentation during labor. The height reached by the fetal presentation during labor was evaluated according to imaging criteria proposed by Dietz et al. The measurement method consisted of the definition of a perpendicular reference to the major axis of the symphysis pubis in the sagittal plane through the lower edge of the pubis, and the measurement of the height of the descent of the lowest point of the fetal head compared to this reference line (A), as well as measuring the difference in height between prelabor and the second stage of labor. The angle of progression was also measured (B). This angle was described by Barbera et al. [9], and represents the angle between a line placed through the midline of the pubic symphysis and a line running from the inferior apex of the symphysis perpendicular to the pubic midline, and to the most anterior part of the fetal skull.

The height reached by the fetal presentation during labor was evaluated according to imaging criteria proposed by Dietz et al. [7] and correlated in MRI by Bamberg et al. [8] (Fig 1).

### Post-processing of 3D imaging

The Predibirth software (Olivier Ami, France) [10,11] was used to perform 3D vector reconstructions. Computer-aided vectorization of the contours was performed, and the results were evaluated by obstetricians and radiologists.

### Statistical analysis

Fisher's exact test was used to test the null hypothesis of independence between fetal descent and the observed changes. The statistical difference between head measurements before and during second stage of labor was assessed using the Wilcoxon signed-rank test.

A P-value less than 0.05 was considered statistically significant. All statistical tests were 2-sided.

## Results

### I) Fetal head molding and brain shape changes

**Fig 2:**
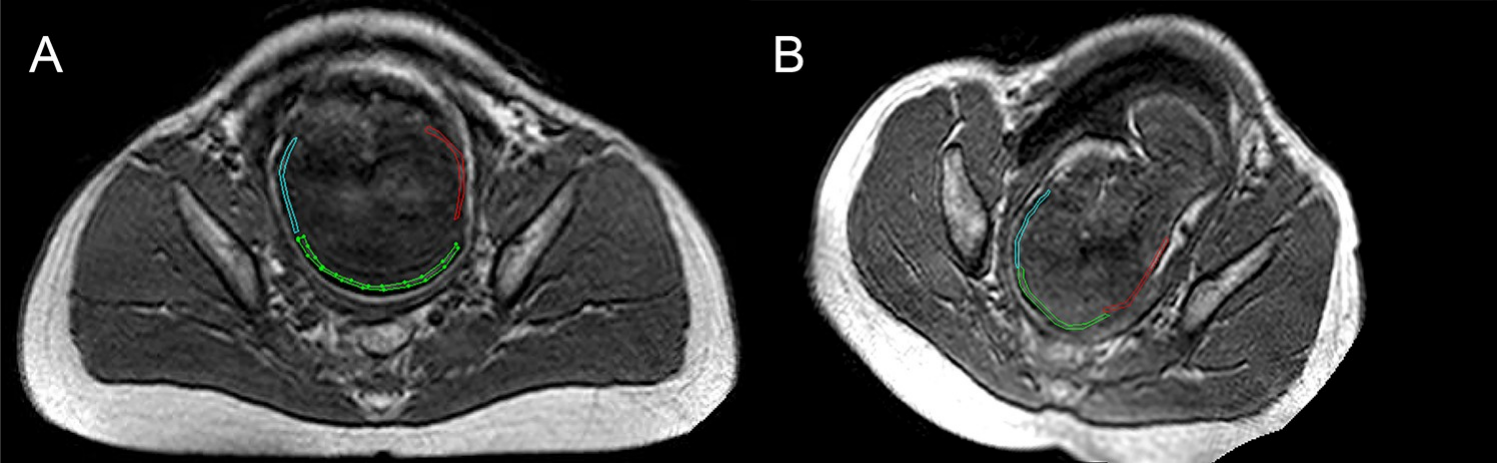
MRI measurement of fetal head molding in patient 1. (a) Axial MRI before entering labor. Finite element splines of the occipital bone (red) and frontal bone (blue) are shown to be continuous with the parietal bone (green). The appearance of the fetal skull is normal. (b) Axial MRI during the second stage of labor. The fetal head is located at the middle of the pelvic brim. The lambdoid fontanelle (seen between the green and red lines) is closed. The parietal bone (green) is shown to slightly overlap the occipital bone (red). There is also slight overlap of the frontal bone (blue) over the parietal bone (green). A characteristic "sugarloaf" deformation of the fetal head was observed on MRI. Similar findings were observed in all fetuses. Suture overlapping was greater in the anterior-posterior direction, especially at the coronal and lambdoid suture, and less important at the sagittal and metopic sutures (Table 1). The head of this fetus kept the “sugar loaf” shape after birth.

The MRI prior to labor did not demonstrate overlapping fetal sutures in any of the fetuses. In contrast, all seven fetuses exhibited fetal head molding with overlapping cranial sutures during the second stage of labor (Figs 1 and 2). Physical examination of the newborns revealed deformed head contours in only two of the seven infants (patients 1 and 7, Table 1), proving the flexibility of the molding system.

**Table 1:** Fetal cranial suture overlap observed on MRI performed during the second stage of labor.

Patients	Metopic suture	characteristic of overlap	coronal suture	characteristic of overlap	sagittal suture	characteristic of overlap	the lambdoid suture	characteristic of overlap	Persistance of molding after birth
**Patient 1**	overlap	frontal right bone below the left frontal bone	no overlap		no overlap		overlap	right parietal bone under the occipital bone	Yes
**Patient 2**	no overlap		overlap	right frontal bone under right parietal bone left frontal bone under left parietal bone	no overlap		overlap	occipital bone under parietal bone	No
**Patient 3**	Overlap	left frontal bone on the right frontal bone	overlap	right parietal bone under right frontal bone	no overlap		overlap	parietal bone under the occipital bone	No
**Patient 4**	no overlap		overlap	right frontal bone under right parietal bone left frontal bone under left parietal bone	overlap	left parietal bone over right parietal bone	overlap	occipital bone over the parietal bone	No
**Patient 5**	no overlap		overlap	right frontal bone under right parietal bone	overlap	right parietal bone over left parietal bone	overlap	occipital bone under parietal bone	No
**Patient 6**	no overlap		overlap	right frontal bone under right parietal bone left frontal bone under left parietal bone	no overlap		overlap	occipital bone under parietal bone	No
**Patient 7**	no overlap		overlap	right frontal bone under right parietal bone	no overlap		overlap	occipital bone under parietal bone	Yes

**Fig 3:**
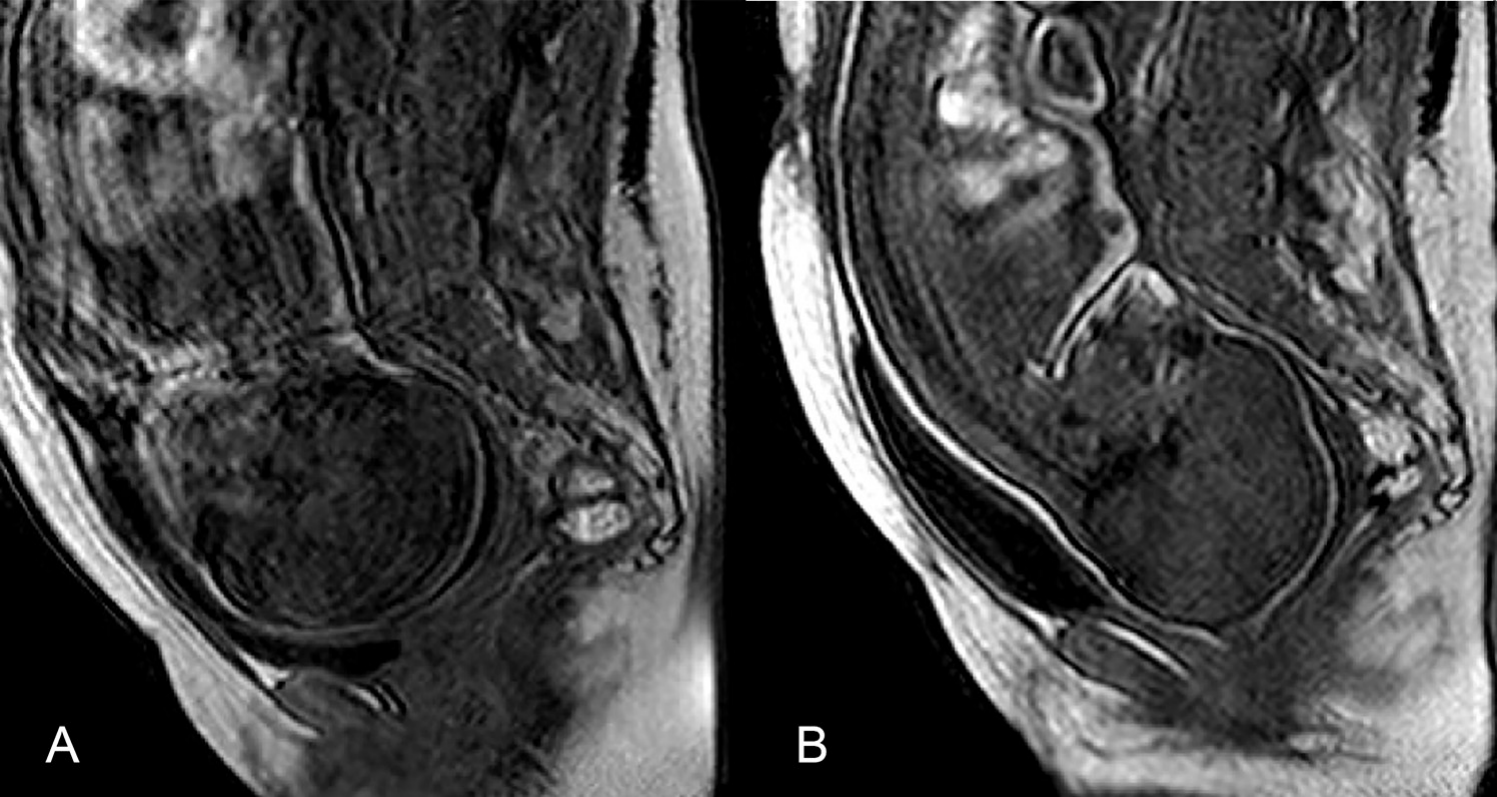
Median sagittal plane MRI before and during labor in patient 5. (a) No fetal head molding was observed before labor. The maternal coccyx was concave anteriorly, and the perineal raphe horizontal.; (b) The fetal head is shown in the middle pelvic brim during the second stage of labor. The fetal presentation has turned to occipito-pubic, the coccyx has tipped backward, and the perineal raphe has assumed a vertical position. The fetal head has typical “sugarloaf molding”. This head molding was not observed after birth.

**Fig 4:**
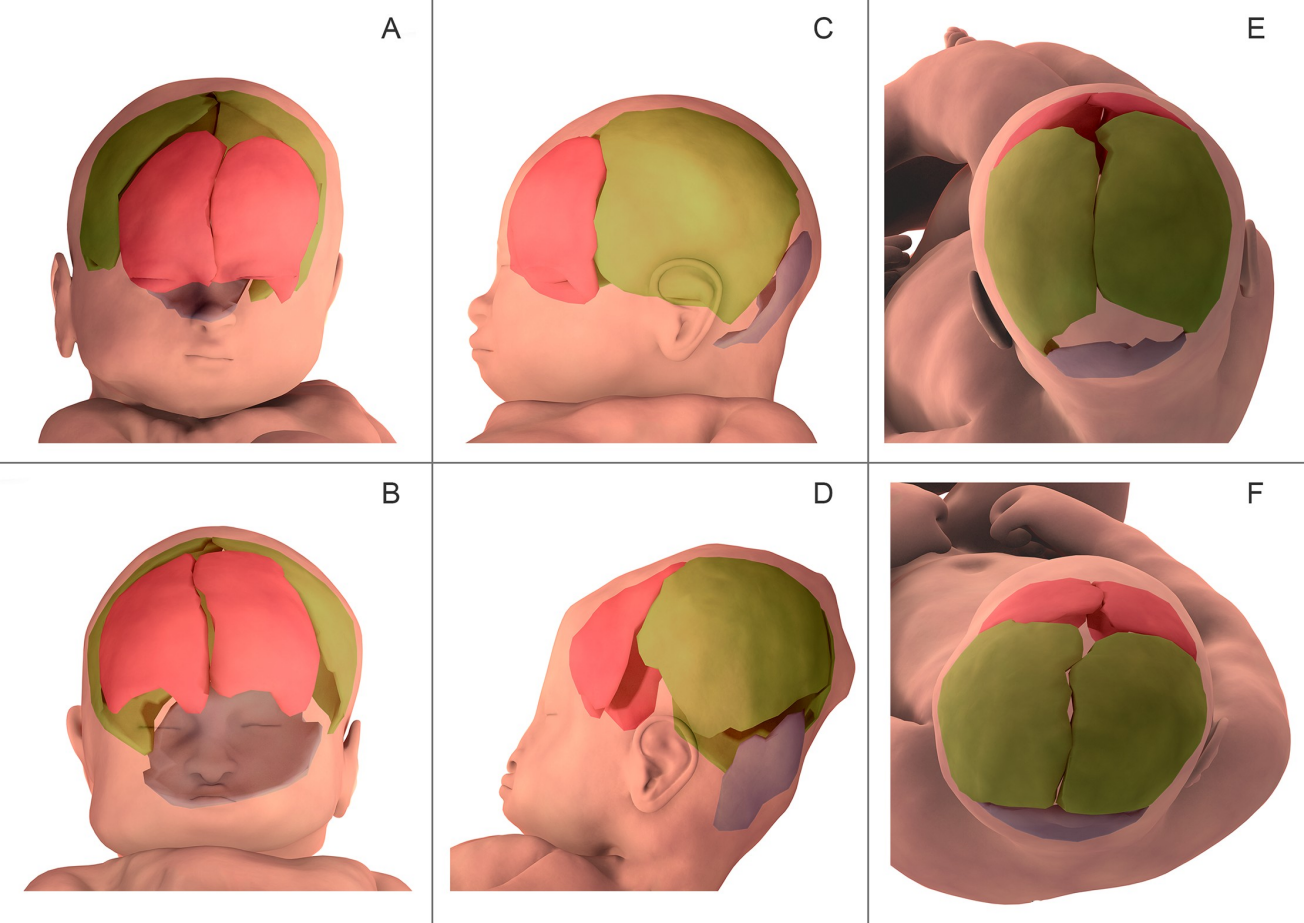
**Three-dimensional finite element reconstruction of the cranial bones before labor (A, C, E) and during the second stage of labor (B, D, F) in patient 5**. Frontal bones are marked in pink, parietal bones in green, and the occipital bone in blue. In the anterior view (A, B) the bregmatic fontanelle was observed to decrease in size during delivery. In the lateral view (C, D) the skull bones were shown to change in relative orientation and amount of overlap. On the superior view (E,F) the bregmatic and lambdoid fontanels were seen to decrease in size during delivery.

Overlapping of the cranial sutures was most marked in the anterior-posterior direction, at the coronal and lambdoid suture. In all cases, at least one of the parietal bones was shifted to a position below the corresponding frontal bone (coronal suture). There was variable shifting of the left and right parietal bones in relation to each other (left below right, n = 1; right below left, n = 4; no overlap, n = 2). Similar variability was observed with overlap of the occipital bone and the parietal bone at the lambdoid suture (Table 1). Together these changes led to the characteristic molding of the bones (Fig 3) and brain (Fig 4) observed at childbirth in the anteroposterior direction, so-called "sugarloaf" head molding.

There was variable shifting of the left and right frontal bones (metopic suture) relative to each other (left below right, n = 1; right below left, n = 3; no overlap, n = 3). Less overlap was observed at the sagittal and metopic sutures (Table 1). No correlation was found between the side of overlap of the metopic and coronal sutures. The bregmatic and lambdoid fontanelles markedly decreased in size in all patients upon entering the middle pelvic brim during the second stage of labor.

The sphenotemporal, sphenofrontal, sphenoparietal, sphenobasioccipital, and occipitomastoid sutures never showed overlap of related bones in any of the seven imaged fetuses. The sphenoid fontanel was enlarged in all fetuses during delivery.

**Fig 5:**
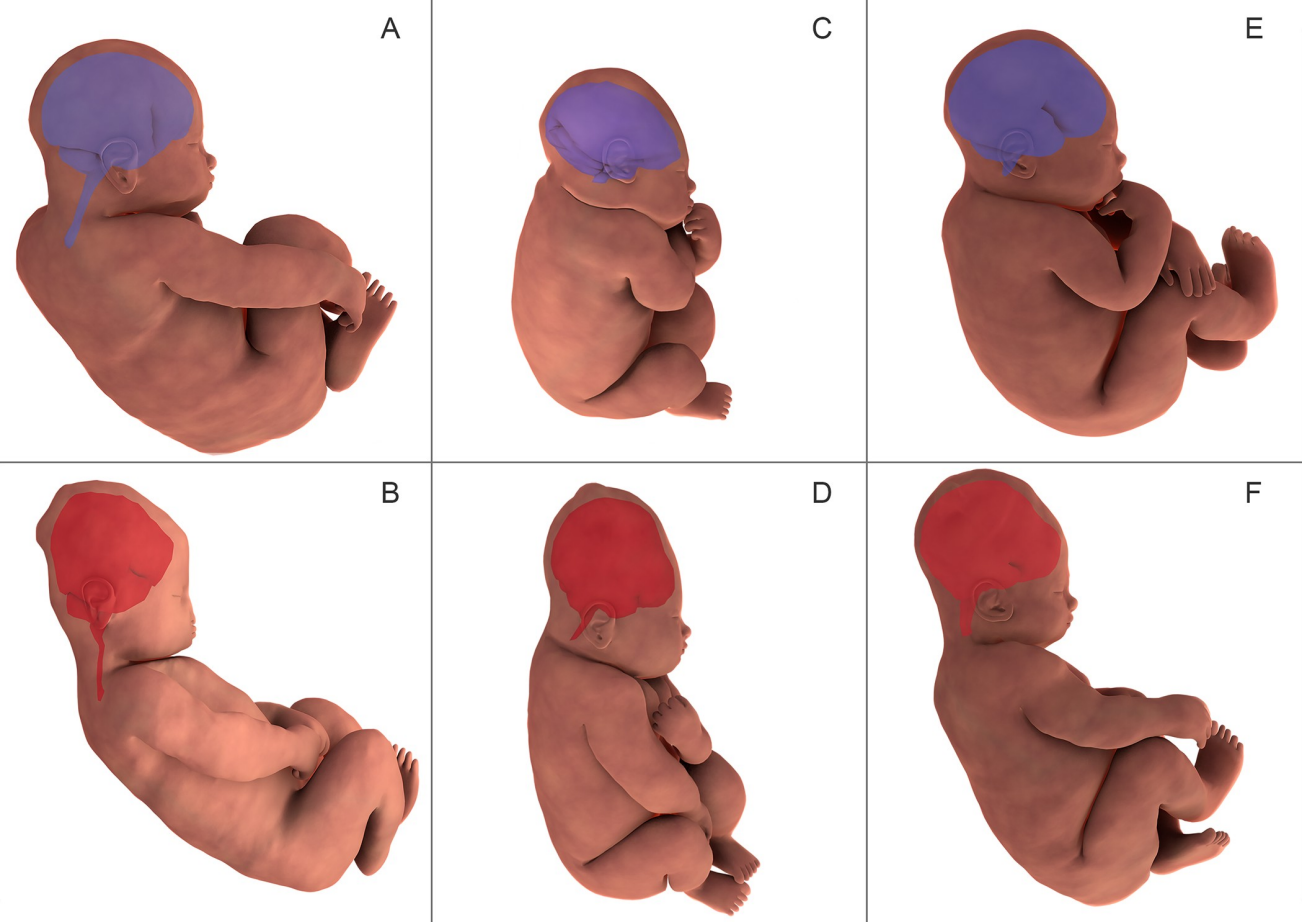
**Three-dimensional fetal brain MRI reconstruction before labor (shown in purple in A, C, E) and during the second stage of labor (shown in orange in B, D, F)**. In all patients, the fetal brain was observed to change in shape in the second stage of labor. This change in fetal brain shape mirrored the shifting of fetal skull bones observed during the second phase of labor.

The relationship between fetal descent and fetal head shape was evaluated. Fetuses during the second stage of labor had a significantly higher incidence of "sugarloaf" deformation of skull and brain than those before labor (Fig 5) (Table 2, Fisher’s exact test, p = .00058).

**Table 2:** Fetal head molding quantitative evaluation during the second stage of labor. FOD: fronto-occipital diameter; Δ FOD: FOD before labor—FOD during second stage of labor (mm); CD: complete dilation. Green color = smaller Δ FODs AND normal delivery; orange color = intermediate Δ FODs AND emergency C-sections; pink color = greatest Δ FOD AND normal delivery.

PATIENTS	DESCENT ACCORDING TO DIETZ IN MM	ANGLE ACCORDING TO DIETZ IN DEGREES	FOD VARIATION BEFORE / DURING IN MM	MAGNIN	CHANGE IN FOD / MAGNIN	DELIVERY TYPE	1 MINUTE APGAR	10 MINUTE APGAR	FETAL BRAIN SHAPE BEFORE LABOR	FETAL BRAIN SHAPE DURING SECOND STAGE
**PATIENT 1**	27.37	21.6	11.64	25.1	0.46	Normal	10	10	Normal	Sugar Loaf
**PATIENT 2**	23.07	17.6	9.23	26.7	0.34	Normal	10	10	Normal	Sugar Loaf
**PATIENT 3**	31.15	26.5	13.21	26.1	0.50	C-section at CD	10	10	Normal	Sugar Loaf
**PATIENT 4**	53.79	48.5	15.16	25.1	0.60	Normal	4	10	Normal	Sugar Loaf
**PATIENT 5**	30.56	24.2	5.66	27.5	0.20	Normal	10	10	Normal	Sugar Loaf
**PATIENT 6**	29.91	26.1	1.83	27.1	0.06	Normal	10	10	Normal	Sugar Loaf
**PATIENT 7**	25.54	25.3	13.31	25.6	0.51	C-section at CD Failed forceps	10	10	Normal	Sugar Loaf

### II) Correlation between imaging findings and clinical outcome

Fetal head molding was observed mostly on the anteroposterior axis (Table 2). The largest change associated with molding was the reduction in fronto-occipital diameter (FOD) from before labor to during the second stage of labor (p = .035, Wilcoxon signed-rank test). This finding correlated with Magnin score and the distance of fetal descent into the pelvis according to the model proposed by Dietz et al. [7]

Patients were selected before delivery on the presumption that if their pregnancies and labors were normal, their deliveries would also be normal. However, two of the seven patients developed clinical cephalopelvic dystocia requiring C-section for delivery (patients 3 and 7, Table 2). One of the patients was found to have the fetal head at the middle of the pelvic brim on clinical exam, while MRI showed the fetal head to be between the superior pelvic strait and the middle brim (patient 7, Table 2). These two fetuses had two of the three greatest FOD reductions from prelabor to the second stage of labor. These findings reinforce the difficulty that can occur when using pelvimetry alone to predict the feasibility of vaginal delivery.

The greatest reduction in FOD (patient 4, Table 2) occurred in the tertipara patient. She had the largest fetus (4525 g) and had a spontaneous vaginal delivery requiring a minimal amount of expulsive effort lasting approximately 15 minutes. Fetal rhythm abnormalities were only observed during the last 15 minutes of expulsion. The 1-minute Apgar score was 4 and the 5-minute Apgar score was 10. The newborn’s umbilical cord pH was 7.29, and lactate levels were 4 mmol/L (normal range: 2.5 to 3.7 mmol/l [12]). These cases show that the degree of head molding shown on MRI alone is not itself predictive of the type of delivery.

Bregmatic and lambdoid fontanelles presented a sharp reduction in their opening at the middle brim in all fetuses during the second phase of labor compared with prelabor. This deformation of the head suggests the shape of a "sugarloaf" (Figs 3 and 4).

## Discussion

### Progress beyond knowledge

Fetal head molding is known to occur sometime during birth, but imaging of this physiological process has only been reported once [13]. All the fetuses imaged here demonstrated some degree of fetal head molding on MRI, but only two of the seven kept a characteristic “sugarloaf”-shaped skull [14] on clinical exam after birth. These findings could be related to different elasticities of the skull bones, the supporting fibrous structure, or the synchondroses at the base of the skull. These MRI findings suggest that the fetus is subjected to greater stress than previously thought, which could explain the high incidence of asymptomatic brain hemorrhages and retinal hemorrhages found after normal vaginal delivery [1,3,15–18]. The fact that one of the children in our series showed the biggest deformation of its skull during the birthing process, was born easily after only a few maternal efforts, and showed signs of fetal distress despite continuous normal monitoring until the expulsion phase, raises the question of the relevance of our actual definition of a normal birth. It is actually considered normal childbirth when a parturient gives birth by natural means, with only a few maternal expulsive efforts. This definition does not take into consideration the ability of the fetal head to deform (compliance). If the fetal head’s compliance is high, the skull and brain may undergo significant deformation as the birth canal is crossed, and the child's condition at birth may not be good. Consequently, childbirth should perhaps be considered normal when a parturient gives birth naturally with only a few expulsive efforts, and her child is fine and has no consequences due to the shape changes of its brain during the process.

### Previous 3D biomechanical assessments of birth

Lapeer et al. [19] had simulated a fetal skull subjected to pressures exerted by the uterine cervix during the first stage of labor using a non-linear model of deformation of the fetal skull. Our study confirmed the molding of the fetal skull that was predicted by the Lapeer biomechanical model, both in terms of its shape after deformation and the degree of deformation. These findings highlight the critical role of cervical dilation on fetal head molding during the first stage of labor. Our findings differed from those of Lapeer et al. in that we observed the parietal bones sliding below the frontal bones. We found no correlation between fetal head rotation inside the birth canal and parietal-frontal bone shifts. Further analysis is required to determine whether there is a correlation between various fetal cranial bone subluxations and the direction of fetal head rotation. Buttin et al. [20] used a spherical model to study deformation of the fetal head during delivery without cervical resistance. This model did not include factors critical to delivery and was not corroborated by our findings.

A literature review found previous studies based on 3D biomechanical simulations of the anatomy of the birthing process; but however, none had been based on imaging involving actual patients and none had demonstrated opportunities for intervention during the process. 3D MRI imaging allowed us to better evaluate biomechanical phenomena occurring during the birthing process and to diagnose the presence of multiple overlaps of the sutures. The evaluation of the degree of fetal head molding by imaging is an important issue to consider in calculating the stiffness modulus of the fetal skull bones and the combined forces which lead to this deformation. These observations allowed us to determine more accurate settings for our computerized delivery simulation models and to better understand the limitations of 3D modeling programs. The Predibirth software (Olivier Ami, France), [10,11] is a 3D post-processing software using MRI, dedicated to studying the process of birth by cephalopelvic confrontation, with libraries of models and fetal behaviors inside the birth canal.

The greatest head molding was observed in two of the three fetuses that required C-section for delivery. It is important to note that even when significant fetal molding occurs, the patient might not have the ability to achieve an uncomplicated birth via vaginal delivery, as found in patient 4. This patient had a Magnin score of 25 and an adequate pelvis for vaginal delivery. However, the newborn had an Apgar score of 4 in the first minute of life, and emitted meconium during the expulsion phase. The Apgar score quickly rose to 10 at 10 minutes of age, and there was no metabolic acidosis. The role of ease of delivery and of fetal molding in this risky clinical presentation is not clear.

### Strengths

To our knowledge, this study is the first time a series of parturient women have been delivered inside an MRI in a reproducible way, which allows us to consider performing further research protocols in the future, with sequences both morphological and functional. MRI modalities allowed us to observe soft tissues that are not correctly visible with ultrasound, and to put into perspective the deformation of the skull, brain, and movement of maternal soft tissues around them. We used high-performance sequences at 1 Tesla to obtain successful imaging in a minimum of time with minimal discomfort, while maintaining a high level of safety, compatible with obstetrics.

### Limitations

For evident safety reasons, we limited our study to physiological labor during this protocol. To explore pathological labor, the MRI machine should preferably be located inside the labor ward. All MRI sequences are not suitable for such protocols, because some take too long to perform. Given childbirth is a dynamic phenomenon, changes occur quickly over time, especially during uterine contractions and releases. Short sequences can be made between contractions, and dynamic sequences can be made on a small number of slices during contractions. The main constraint was the availability of a dual team of maternity (obstetrician, pediatrician, anesthetist, midwife, pediatric nurse) and radiology (radiologist, radio manipulator) 24 hours a day, including a team dedicated to the protocol. We did not observe any fetal brain impairment during or after delivery because the protocol did not include diffusion sequences to detect ischemia or T1 FATSAT to observe the onset of hemorrhage.

## Conclusion and future studies

This study demonstrates the value of 3D MRI assessment using 3D finite elements mesh reconstruction during the second stage of labor to reveal how the fetal brain is impacted by the molding of the cranial bones. Fetal head molding was observed on MRI in all seven patients during the second phase of labor. The cranial deformation observed on MRI was not observed on clinical exam in five of the seven newborns. Overlapping of the cranial sutures was most marked in the anterior-posterior direction, at the coronal and lambdoid suture. These findings need to be confirmed in a larger series.

The observations made on the modeling capabilities of the fetal head and brain during the second stage of labor also allow us to consider a more realistic simulation of delivery in order to develop a virtual trial of labor that can detect and prevent biomechanical risks linked to childbirth.

## Supporting information

S1 dataset "RESULTS_imaginaitre_plosone.xlsx" contains the minimal data set for the manuscript results presented here. (XLSX)
